# Long-Term Outcome after Vitrectomy for Macular Edema with Retinal Vein Occlusion Dividing into the Occlusion Site

**DOI:** 10.1155/2014/198782

**Published:** 2014-10-13

**Authors:** Takeshi Iwase, Brian C. Oveson

**Affiliations:** ^1^Department of Ophthalmology, Nagoya University Graduate School of Medicine, Nagoya University Hospital, 65 Tsuruma-cho, Showa-ku, Nagoya, Aichi 466-8560, Japan; ^2^Department of Ophthalmology, Toyama Prefectural Central Hospital, Toyama, Japan; ^3^Department of Ophthalmology, Johns Hopkins University School of Medicine, Baltimore, MD, USA; ^4^State University of New York at Stony Brook, Stony Brook University Hospital and Medical Center, Stony Brook, NY 11794-8223, USA

## Abstract

*Purpose.* To investigate the efficacy of treatment for macular edema secondary to retinal vein occlusion (RVO) with vitrectomy. *Methods.* This retrospective study identified patients with macular edema associated with RVO between January 2004 and April 2006. Inclusion criteria were eyes with (1) preoperative visual acuity (VA) of 20/40 or worse, (2) a central foveal thickness (CFT) greater than 250 *μ*m, and (3) vitrectomy with internal limiting membrane and intravitreal triamcinolone acetonide. Each patient had their RVO classified as a major or macular BRVO or hemispheric RVO (HSRVO). *Results.* Forty-six eyes with major BRVO, 18 eyes with macular BRVO, and 17 eyes with HSRVO were investigated. VA was significantly improved at 24 months after surgery for each group (*P* < 0.05). Vision in the macular BRVO group 24 months after surgery was significantly better than that in other groups (*P* < 0.05). For each group, a concomitant reduction of CFT was noted at every time point when compared to preoperative values (*P* < 0.001). *Conclusions.* In macular BRVO, the postoperative vision 24 months after surgery was significantly better than the other groups. These findings suggest that additional and earlier treatments might be more important for patients with major BRVO and HSRVO than for those with macular BRVO.

## 1. Introduction

Branch retinal vein occlusion (BRVO) is the second most frequent major retinal vascular disease after diabetic retinopathy [1]. Previous population-based studies have reported prevalence rates for BRVO ranging from 0.3% [2] to 1.1% [3]. BRVO most commonly occurs at sites where arterioles cross over veins, and pathologic findings support the hypothesis that thickening of the arteriole wall compresses the lumen of the retinal vein, thus altering flow and promoting thrombosis [4–6]. BRVO consists of 2 distinct clinical entities: major BRVO and macular BRVO [7, 8]. A third type, hemispheric retinal vein occlusion (HSRVO), has been characterized as an occlusive process involving a hemiretina. Venous obstruction occurs at an arteriovenous crossing located on the optic disc, or close to the optic disc, sharing the same pathogenesis with major BRVO [1, 9, 10]. The severity of BRVO varies depending upon the location of the occlusion; in general, the more proximal the occlusion, the more severe the edema. Although it is important in the clinical management to classify RVO into subtypes based on the location of the occlusion, not many studies provided that information [11–15]. The long-term outcomes for vitrectomy-treated RVO cases have not been sufficiently reported, and further investigations are needed. In order to improve treatment for RVO patients, this study divided RVO into 3 main groups: major BRVO, macular BRVO, and HSRVO and investigated the outcomes after surgery.

In recent decades, a variety of treatment modalities have been explored, including laser photocoagulation, intraocular corticosteroid injections, intravitreal injection of antivascular endothelial growth factor (VEGF), and arteriovenous sheathotomy with or without pars plana vitrectomy (PPV) [16–21]. Anti-VEGF therapy improves visual acuity (VA) and has become a gold standard recently, but it requires frequent intravitreal injection to maintain a stable vision and can have complications [22].

To the best of our knowledge, there has not been a report describing the 2-year outcomes of vitrectomy-treated RVO patients that were compared based on location of the occlusion. Thus, the purpose of this study was to investigate the efficacy of the treatment for macular edema secondary to RVO with concomitant PPV, internal limiting membrane (ILM), and intravitreal triamcinolone acetonide (TA) for two years after differentiating the cases into the following 3 main groups according to the site of venous occlusion: major BRVO, macular BRVO, and HSRVO.

## 2. Patients and Methods

This single-center retrospective study was conducted at the Department of Ophthalmology, Toyama Prefectural Central Hospital. Surgery was carried out based on the approval of the institutional review board and the ethical standards established by the Declaration of Helsinki and informed consent was obtained from all patients.

### 2.1. Patients

We retrospectively reviewed patient records to identify patients who had macular edema secondary to BRVO between January 2004 and April 2006. Inclusion criteria were (1) preoperative visual acuity of 20/40 or worse, (2) macular edema secondary to BRVO with observation over 3 months, (3) a central foveal thickness (CFT) greater than 250 *μ*m, and (4) vitrectomy with ILM and intravitreal TA. Exclusion criteria were vitreous hemorrhage, severe cataract, epiretinal membrane, history of vitreoretinal surgery, and other ocular diseases that could contribute to visual loss. Also, patients with a history of previous macular laser photocoagulation were excluded from the study. The patients were divided into 3 groups according to the site of venous occlusion: major BRVO (occlusion of one of the major branch retinal veins), macular BRVO (occlusion of one of the macular venules), and HSRVO.

Each patient underwent a detailed preoperative evaluation, including best-corrected VA measurement, slit-lamp biomicroscopy, indirect ophthalmoscopy, fundus photography, fluorescein angiography (FA), and optical coherence tomography (OCT). The following patient information was also collected: age, gender, axial length, lens status, and intraocular pressure (IOP). Postoperatively, ocular examinations were performed on 1 week and 1, 3, 6, 12, and 24 months after surgery. All patients were followed up for more than 12 months after surgery. Snellen VA was converted to the logarithm of the minimum angle of resolution (Log MAR) units to create a linear scale of VA.

### 2.2. Surgical Procedure

All surgeries were performed by a single surgeon (T.I.). On phakic eyes, to avoid decreased VA due to cataract progression, concurrent phacoemulsification with an implantation of intraocular lens was performed, followed by standard 20-gauge three-port PPV. After core vitrectomy, posterior vitreous detachment was created if the cortical vitreous was adherent to the retina. The ILM was peeled off with the assistance of TA around the macula, and the diameter of the ILM-removed area was approximately 2-3 disk diameters centered on the fovea. Next, peripheral scatter laser photocoagulation was performed to prevent neovascularization in major BRVO and HSRVO groups. Finally, 4 mg of TA was injected into the vitreous cavity just before the sclerostomy sites were closed.

### 2.3. Statistical Analysis

Normality of data distribution was assessed using the Kolmogorov-Smirnov test. Differences that showed a normal distribution were compared using the unpaired Student's* t-*test. Continuous variables without a normal distribution were compared using the Mann-Whitney* U* test. A repeated-measures analysis of variance was used to compare differences at the various examinations. Differences with a *P* value less than 0.05 were considered statistically significant.

## 3. Results

### 3.1. Baseline Characteristic and Patient Disposition

A total of 114 patients had macular edema associated with BRVO and HSRVO at the first visit in the period. There were 64 eyes in the major BRVO group, 30 eyes in the macular BRVO group, and 20 eyes in HSRVO group.

After more than 3 months of follow-up without any treatments, a total of 81 eyes of 81 patients still had macular edema and underwent the surgery and were included in the present study. There were 46 eyes in the major BRVO group, 18 eyes in the macular BRVO group, and 17 eyes in HSRVO group. The average age in the HSRVO group was significantly higher than the other groups (*P* = 0.015) (Table 1). The preoperative VA was 0.64 ± 0.33 in the major BRVO group, 0.54 ± 0.23 in the macular BRVO group, and 0.65 ± 0.28 in the HSRVO group, and there was no significant difference in the VA among the groups. The preoperative CFT was 618 ± 209 in the major BRVO group, 558 ± 153 in the macular BRVO group, and 730 ± 269 in the HSRVO group, and there was no significant difference among the groups. In addition, there were no significant differences in gender, axial length lens status, IOP, or location of occlusions, for example, superior or inferior site (Table 1).

### 3.2. Function Outcomes after Surgery

**Figure 1:**
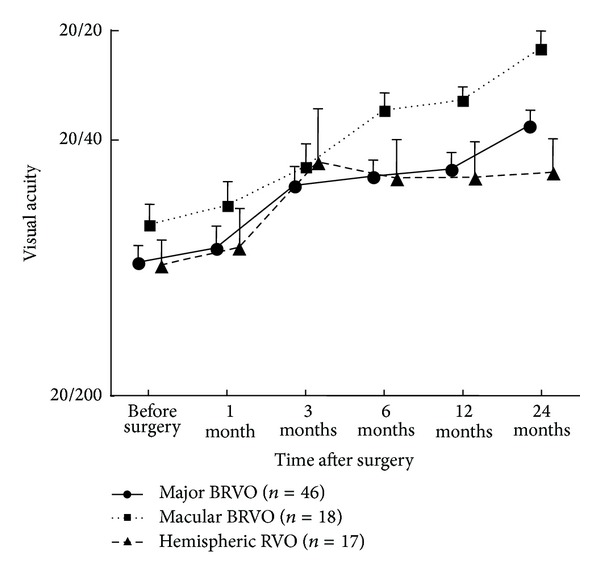
Best-corrected visual acuity during the follow-up period. Visual acuities are plotted in logarithm of the minimal angle resolution unit. The postoperative visual acuity was gradually increased and significantly improved in all of the groups in 24 months after surgery. The visual acuity 24 months after surgery in the macular BRVO group is better than the other groups (*P* < 0.05).

Figure 1 shows the improvement in VA after surgery. The vision was gradually increased and significantly improved to 0.31 ± 0.31 in the major BRVO group (*P* < 0.001), 0.16 ± 0.20 in the macular BRVO group (*P* < 0.001), and 0.39 ± 0.43 in the HSRVO group (*P* = 0.048) at 24 months after surgery. The postoperative VA at 24 months after surgery in the macular BRVO was significantly better than that in other groups (*P* < 0.05).

**Figure 2:**
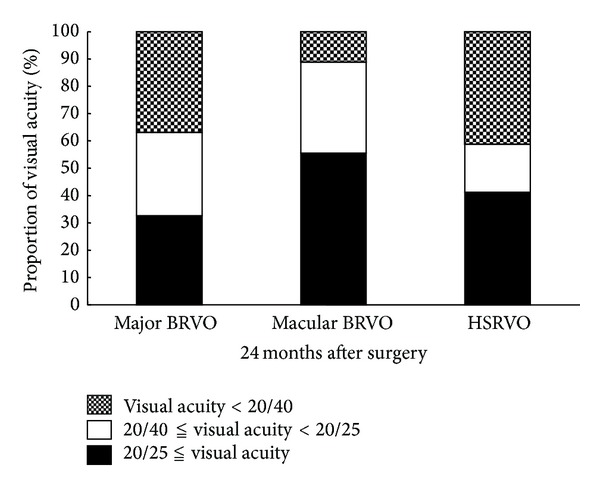
The proportion of visual acuity in each group. There is no significant difference in the proportion among the groups.

At 24 months after surgery, the proportion of patients who had ≧20/25 vision was 33% in the major BRVO, 56% in the macular BRVO, and 41% in the HSRVO. The proportion of patients who had <20/40 was 37% in the major BRVO, 11% in the macular BRVO, and 41% in the HSRVO. There were no significant differences in the proportion of the final VA among the groups (Figure 2).

### 3.3. Anatomic Outcomes after Surgery

**Figure 3:**
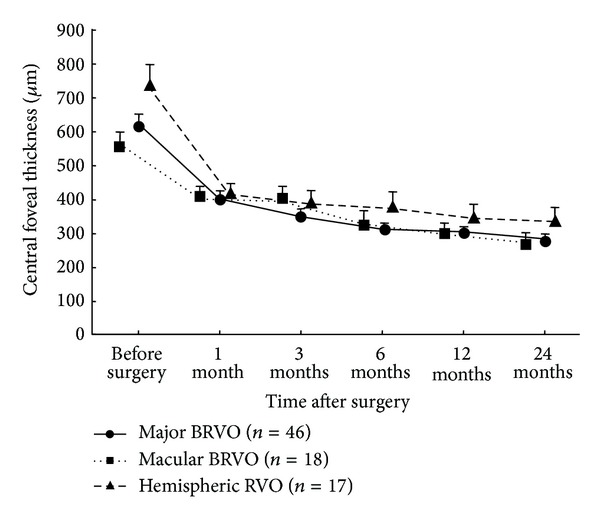
Central foveal thickness (CFT) during the follow-up period. The CFT was reduced significantly in each of the groups at every time point after surgery compared with before surgery (*P* < 0.001).

**Figure 4:**
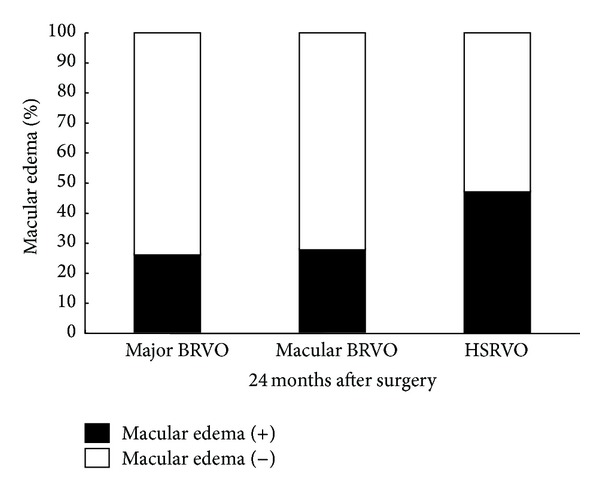
The proportion of macular edema in each group. There is no significant difference in the proportion among the groups.

Concomitant with the improvement in VA, there was a significant reduction in CFT in each of the groups at every time point after surgery when compared to the values before surgery (*P* < 0.001) (Figure 3). One month after surgery, the mean reduction from baseline CFT was 225 *μ*m in the major BRVO group, 187 *μ*m in the macular BRVO group, and 321 *μ*m in the HSRVO group. The CFT was gradually decreased over time within each group. The mean CFT was 278 *μ*m in the major BRVO group, 267 *μ*m in the macular BRVO group, and 329 *μ*m in the HSRVO group 24 months after surgery. There was no significant difference in CFT among the groups at any time point after surgery. The percentage of patients whose macular edema had resolved at 24 months after surgery was 74%, 73%, and 53% of major BRVO group, macular BRVO group, and HSRVO group, respectively, and there was no significant difference in the proportion of the resolution among the groups (Figure 4).

## 4. Discussion

In this study, we determined treatment efficacy for RVO patients that were divided into 3 main groups according to the site of venous occlusion: major BRVO, macular BRVO, and HSRVO. The severity of major BRVO and HSRVO depends on the vessel occluded and can involve a full range of complications. On the other hand, patients with macular BRVO are unlikely to develop neovascularization because a smaller area of the retina is affected; however, they frequently suffer from macular edema and visual impairment. Therefore, distinguishing RVO cases based on location of venous occlusion has significant bearing on long-term patient outcomes.

BRVO usually has a favorable natural history as approximately 60% of patients retain 20/40 vision or better [1]. Recently, Hayreh and Zimmerman reported that the macular edema resolved in 56% of major BRVO cases and in 60% of macular BRVO cases without treatment over a 24-month follow-up and approximately 80% of BRVO cases after 60-month follow-up [12]. In the present study, 24 months after surgery, macular edema had resolved in 74%, 73%, and 53% of the major BRVO group, macular BRVO group, and HSRVO group, respectively, and there was no significant difference in the resolution among the groups. In the present study, we cannot exclude the possibility that macular edema may have resolved and vision improved without surgical intervention. In our study, a 3-month observation period prior to surgery was useful in order to exclude a large proportion of patients with macular edema that would spontaneously improve without surgical intervention. In our study, the CFT had significantly decreased in all of the groups just one month after surgery and remained low throughout this 24-month study. The results suggest that vitrectomy with ILM peeling and intravitreal TA is an effective treatment for macular edema caused by BRVO and HSRVO in terms of improving foveal morphology.

Anti-VEGF therapy improves VA and has become a gold standard recently, but it requires frequent intravitreal injection to maintain a stable vision and can have complications [22]. It is well known that impairment of macular function is caused by the longstanding presence of macular edema or ischemia in patients with BRVO. Outer retinal diseases with edema, such as neovascular age-related macular degeneration, cause rapid and often irreversible vision loss secondary to damage to the critical photoreceptors in a relatively short period of time. Therefore, surgical intervention may be necessary in order to achieve favorable functional outcomes, especially for the eyes with refractory macular edema for anti-VEGF therapy. One possible mechanism for the resolution of macular edema by vitrectomy is improvement of the oxygen supply to the ischemic inner retina [23]. Another mechanism is that vitrectomy could remove any traction on the retinal surface by removing the posterior vitreous cortex [24]. Internal limiting membrane removal may also be beneficial [25]. In the present study, TA was injected intravitreously during surgery and may have played a role in resolving the macular edema [21]. Numerous studies in the literature have demonstrated promising outcomes with intravitreal TA treatment for macular edema due to BRVO [26–29]. Although steroids have antiangiogenic, antifibrotic, and antipermeability properties, the principal effects of steroids are stabilization of the blood-retinal barrier, resorption of exudation, and downregulation of inflammatory stimuli [30].

An optimal outcome is good vision with resolved edema in the treatment for RVO. In this study, the postoperative VA had improved significantly in all of the 3 groups during the postoperative observation. In macular BRVO, the postoperative vision 24 months after surgery was significantly better than the other groups. On the other hand, the CFT values showed that there were no differences in resolution of macular edema and reduction of the CFT among the groups. Noma et al. reported that vitreous fluid levels of VEGF and inflammatory factors were higher in the major BRVO group than the macular BRVO group which may have affected the preoperative VA and the CFT, though there was no significant difference in the postoperative status at 6 months after surgery [14]. Our results were different from their results regarding the postoperative vision, though there was no significant difference in the VA at 6 months after surgery in the present study as well. One of the reasons is that we followed up the patients for 24 months after surgery. The damage to the critical photoreceptors at the fovea with preoperative higher VEGF and lower anti-inflammatory factors might affect postoperative visual acuity in BRVO and HSRVO in long-term follow-up.

Venous obstruction in HSRVO occurs at an arteriovenous crossing located on the optic disc, or close to the optic disc, sharing the same pathogenesis with major BRVO [1, 9, 10]. To the best of our knowledge, however, there have not been reports demonstrating the comparison between major RVO and HSRVO. Interestingly, there was no significant difference in pre- and postoperative VA, CFT, or other factors, except for age between the groups. In most eyes, BRVO occurs in the temporal retina rather than the nasal retina because of the higher number of arteriovenous crossing sites [31, 32]. The major BRVO group in this study consisted of obstruction at the temporal area. It was reported that vitreous fluid levels of inflammatory factors are correlated with the nonperfusion area of the retina [13]. The obstructed area in HSRVO includes the nasal retina, which is smaller than the temporal retina. Our results might suggest that the difference between the obstructed area of major BRVO and HSRVO is smaller than the difference between macular BRVO and the other groups. This observation may distinguish the final visual acuity differences between the subsets of RVO described in this study.

The present study had the following limitations. First, although the VA improved in all of the groups, some cases retained macular edema, most notably were the eyes with HSRVO in this study. The results suggest that vitrectomy with ILM peeling and intravitreal TA may not be sufficient to meet the optimal goal for every patient with RVO. Pre- and postoperative retinal ischemia should affect the postoperative VA. Another important limitation of this study was that we did not measure vitreous fluid levels of VEGF pre- and postoperatively and did not follow up the area of the retinal ischemia using FA. These factors made it difficult to determine the change of the retinal ischemia after surgery, especially at the fovea, though the macular edema was followed up with OCT.

## 5. Conclusion

Vitrectomy with ILM peeling and intravitreal TA injection improved macular edema caused by major and macular BRVO and HSRVO compared to baseline measurements in patients that initially showed no improvement during a three-month observation period prior to surgical intervention. Although there were no differences in resolution of macular edema between the groups 24 months after surgery, the visual acuity for the macular BRVO group was significantly better compared to the other groups. These findings suggest that another earlier treatment might be more important for patients with major BRVO and HSRVO than for those with macular BRVO.

## Figures and Tables

**Table 1:** Baseline characteristic and patient disposition.

Parameter	Major BRVO (*n* = 46)	Macular BRVO (*n* = 18)	Hemispheric RVO (*n* = 17)	*P* value
Age (years)				
Mean ± SD	65.7 ± 11.6	68.8 ± 10.4	74.6 ± 8.0	0.015
Median	64	69	76	
Range	51–91	54–88	56–90	
Gender (*n*)				
Male	28	8	10	0.482
Female	18	10	7	
Site of venous occlusion (*n*)				
Superior	34	13	10	0.489
Inferior	12	5	7	
BCVA				
Log MAR	0.64 ± 0.34	0.54 ± 0.23	0.65 ± 0.28	0.435
Median	20/70	20/70	20/100	
Range	20/250–20/50	20/200–20/50	20/500–20/50	
IOP (mmHg, mean ± SD)	15.5 ± 3.5	16.7 ± 4.3	15.6 ± 3.9	0.364
AXL (mm, mean ± SD)	23.61 ± 1.1	23.46 ± 1.1	23.11 ± 1.7	0.127
Lens status *n* (%)				
Phakia	42 (91%)	17 (94%)	16 (94%)	0.878
CFT (*μ*m, mean ± SD)	618 ± 209	558 ± 153	730 ± 269	0.080

BCVA: best-corrected visual acuity; IOP: intraocular pressure; AXL: axial length; CFT: central foveal thickness.
